# The Hot Compressed Air Treatment of Diabetic Gangrene

**Published:** 1908-05-23

**Authors:** 


### Points in Treatment.

### THE HOT COMPRESSED AIR TREATMENT OF DIABETIC GANGRENE.

The difficulties met with in the treatment of diabetic and other forms of gangrene of the toes and feet are well enough known to require no elaboration here. Surgical measures may succeed in checking the spread of the mischief; but even amputation is fraught with the gravest anxiety, for the reparative powers of the tissues above the site of actual gangrene are often so defective that septic processes and further gangrene are very apt to recur at the site of operation even when the latter is far up the leg.

There is, however, a method of treatment which is much better known upon the Continent than it is in this country, but before describing it, let it not be forgotten that the treatment of each patient must be decided upon the merits of the particular case: in some instances the ordinary well-known methods require no assistance; in others, however, any additional means of benefiting the dire condition of the patient are welcome. It is in the latter cases that a knowledge of the hot compressed-air treatment may be of service. Nor is it only in cases of diabetic gangrene that it may be used; gangrene from other causes, sloughing new growths and other similar lesions may benefit from it also.

The procedure appears to have been devised in the first instance by M. Prat at Lyons in 1889, though Hollander, of Berlin, also worked upon the same lines, and Bier has devised an allied, but by no means identical, method. If hot air alone sufficed to do what was wanted, no particular apparatus would be needed; but from the comparative results that have been published it is quite clear that relief or cure is very much influenced by having the air not only hot, but also at a pressure greater than that of the atmosphere. The apparatus devised and extensively used by M. Prat and others is such that compressed air is allowed to play directly upon the part to be treated, and the heating of the air is carried out by means of platinum wires kept incandescent by a current of carburetted vapour. There is an ingenious device by means of which the intensity of the heat or the pressure, or both, can be varied at will, and it is most important that this should be so owing to the varying requirements of different cases. M. Prat's method differs in this respect from that of Bier; both cause active hyperæmia of the part, but Prat's goes further, and actually inhibits the rate of multiplication of the microbes present. This seems to be the essence both of the interest and of the utility of the method.

Applied to parts which are already painful, hot compressed-air at a temperature of 150° C. to 300° C. would in some cases be unbearable without a general anæsthetic; in others, on the other hand, no chloroform is required. After the application warm fomentations of sterilised water applied to the part and frequently renewed relieve the pain in a very short time. The fact that an anæsthetic may be needed is by itself good evidence that the method will only be employed in special cases. This, however, is no good ground for putting the idea of it on one side at once, for there are plenty of cases, even amongst wealthy patients, in whom almost anything would be given for a means of checking gangrene. By the hot compressed-air treatment upon the Continent, the rapidity with which the stench of a gangrenous part is stopped is almost remarkable. It is needless to quote many cases; one of a series reported by MM. René Bonamy, Marot, and Vignat in Paris* will suffice to indicate the manner in which the treatment acts:—

A patient aged 65 came into hospital on January 17 for moist gangrene of the fourth toe of the left foot, the result of an injury. On January 23, without either general or local anæsthetic, the gangrenous toe was disarticulated and removed. The same evening the patient's temperature rose to 100.8° F., and it remained up for several days. The man had a cough, with non-consonating râles at the bases of his lungs. His general condition became very grave, and he was semi-delirious. On January 27 the left foot had swollen up to double its previous size; it had a fœtid smell, and its colour was a peculiar reddish-violet. The skin of the whole of the plantar surface was puffed up by an enormous dark blister, which when opened discharged a thin, dark, stinking fluid mixed with fœtid gas. There were crepitations of subcutaneous emphysema in the tissues around the absolutely gangrenous part. A diffuse subcutaneous infiltration rapidly spread through between the fourth and fifth metatarsal bones to the dorsum of the foot, thence creeping up the leg; the latter became covered with dark blisters and blebs.

The twenty-four hours' urine at this time contained 45 grains of albumen, 2,490 grains of sugar, traces of acetone, and less urea than normal. The patient seemed to be *in extremis*. Dr. Bonamy and his colleagues report that at this moment Dr. Vignay brought in M. Prat, who demonstrated his apparatus. It was clear that no other measures for saving the patient were possible, and leave was granted to him to do his best for an apparently dying patient. The first application of the hot compressed-air was made forthwith, without any anæsthetic. The fomentations were removed, and a douche of the hot compressed-air was allowed to play all over the affected parts. Under the influence of this the tissues could be seen to become drier, to retract, and to become darkened as though carbonised. The patient did not seem to suffer from this application so long as it was confined to the diseased parts, but he felt pain directly the healthy tissues were reached. Very hot fomentations were ordered. Next day a second application was given. The stench was great when the fomentations were removed, and there was much oozing, but the actual gangrene had not advanced. On January 29 the extent of the gangrene had remained unchanged, but the gangrenous parts had

* "Bulletins et Mémoires de la Société médicale des Hôpitaux de Paris," 1908, p. 521.

become depressed below the general surface, the plantar aponeurosis was visible, and the blebs upon the healthy skin were quietening down, leaving rose-red centres with a margin of white tissue round them.

On January 30 those who saw the case were obliged to admit that the spread of the gangrene had been completely checked since the treatment had been adopted.

By February 1 the patient was beginning to feel the application more and more painful. The odour of the gangrene had almost entirely gone. By February 6 the volume of the foot had become practically normal. The wound had completely ridded itself of all sloughs, and its surface was covered by red granulations.

By April 3 the site of the gangrene had become almost entirely covered with fresh epidermis. There was still a granulating spot about a quarter of an inch in diameter, but the rest of the wound had regained an almost normal appearance, and the new-formed scar-tissue was smooth and supple. The patient was discharged from the hospital with a dressing upon his foot, but able to walk.

For lack of space we cannot give three other very similar cases recorded from the same source, and all treated in a similar way. It will be clear that there are uses for the hot compressed-air method of treatment. In the case quoted not only was the patient much too ill to have amputation performed, but also by the alternative treatment he was enabled to leave the hospital still the possessor of two legs. Such an advantage is so great that one would like to hear of other cases treated in the same way. The hot compressed-air method of treatment in these cases seems well worthy of trial in this country.

